# Estimation of Starch and Sugar Intake in a Japanese Population Based on a Newly Developed Food Composition Database

**DOI:** 10.3390/nu10101474

**Published:** 2018-10-10

**Authors:** Aya Fujiwara, Kentaro Murakami, Keiko Asakura, Ken Uechi, Minami Sugimoto, Han-Chieh Wang, Shizuko Masayasu, Satoshi Sasaki

**Affiliations:** 1 Department of Social and Preventive Epidemiology, Graduate School of Medicine, University of Tokyo, Tokyo 113-0033, Japan; fujiwaraay-tky@umin.ac.jp (A.F.); msugimoto@m.u-tokyo.ac.jp (M.S.); hanchieh-w@m.u-tokyo.ac.jp (H.-C.W.); 2 Department of Social and Preventive Epidemiology, School of Public Health, University of Tokyo, Tokyo 113-0033, Japan; kenmrkm@m.u-tokyo.ac.jp; 3 School of Medicine, Toho University, Tokyo 143-8540, Japan; keiko.asakura@med.toho-u.ac.jp; 4 Faculty of Health Science, Toho University, Chiba 274-8510, Japan; ken.uechi@hs.toho-u.ac.jp; 5 Ikurien-Naka, Ibaraki 311-0105, Japan; masayasu@ikurien.com

**Keywords:** starch, sugar, saccharide, Japan, epidemiology, dietary survey

## Abstract

Due to a lack of a food composition database on starch and sugars, we developed a comprehensive database on starch and seven types of sugars in commonly consumed foods (*n* = 2222) in Japan. Dietary record data of 368 toddlers (aged 18–35 months), 376 preschool children (aged 3–6 years), 915 schoolchildren (aged 8–14 years) and 392 adults (aged 20–69 years) were used. The mean starch intake ranged from 55.6 g/day (female toddlers) to 206.0 g/day (male schoolchildren). Irrespective of age and sex, >50% of starch was provided by rice and grains. The mean total sugar intake ranged from 46.1 g/day (female toddlers) to 68.7 g/day (male schoolchildren). In all age and sex groups, the major contributors of total sugar included sucrose (mean: 18.2–34.0 g/day), glucose (7.8–13.1 g/day), lactose (5.3–13.1 g/day) and fructose (7.6–11.1 g/day). The top food sources were dairy products (toddlers) and confectionaries (other age groups) for total sugar, confectionaries for sucrose, fruits (toddlers) and vegetables (other age groups) for glucose, dairy products for lactose and fruits (toddlers and preschool children) and vegetables (schoolchildren and adults) for fructose. In conclusion, this study clarified the starch and sugar intake in Japan and provides a foundation for future research.

## 1. Introduction

Much attention has recently been given to the potential influences of dietary carbohydrates on human health [1]. In particular, the intake of sugars that are added during manufacturing and cooking (namely added or free sugar) is under scrutiny by the World Health Organization (WHO) and many countries [1,2] because of positive associations with an increased risk of dental caries [3], obesity [4], type 2 diabetes [5], cardiovascular diseases [6,7] and pancreatic cancer [8]. In contrast, potential associations between starch and saccharides (such as sucrose, lactose, maltose, trehalose, glucose, fructose and galactose) and health status have not been completely investigated or are controversial [8,9]. However, the effects on health could possibly differ between these carbohydrate subtypes. For example, sucrose is considered the most cariogenic carbohydrate, whereas starch, galactose and trehalose are lesser cariogenic than sucrose [10,11]. With regard to type 2 diabetes, the associations with carbohydrate subtypes (including starch, total sugar, sucrose, fructose, glucose, lactose and maltose) are not consensual in prospective cohort studies [12,13,14,15,16,17,18,19,20,21,22]; for example, while total sugar intake has been shown to be inversely associated with the risk among Australian adults [13], there was no association in other studies [19]. Among saccharides, fructose intake is suggested to mechanistically contribute to non-alcoholic fatty liver disease (NAFLD), dyslipidemia and insulin resistance, through gluconeogenesis and hepatic de novo lipogenesis [23,24], although fructose intake did not lead to NAFLD [25] or increase triglyceride levels [26] in several controlled feeding trials.

Before investigating the health effects of these carbohydrates, it is essential to develop a method for assessing these carbohydrates. In Western countries, the intake of starch [27,28,29,30,31], sucrose [32,33,34] (which is often considered a surrogate of added sugar rather than a certain disaccharide) and fructose [33,34,35,36] have been widely reported, while less information on the intake of other saccharides is available [18,33,34]. In contrast, little is known about the intake of total, added, or free sugar, starch and saccharides in Asian countries, including Japan [37,38,39]. This is mainly due to the lack of a comprehensive composition database on starch and sugars. Recently, the starch and saccharide contents in food items were reported for the first time in the Standard Tables of Food Composition in Japan 2015 (STFCJ) [40,41]. Nevertheless, only 40% of food items included had starch and saccharide contents and data on total and free sugar were not available. Generally, Japan has a high consumption of rice, soybean products, vegetables, fish, green tea and seasonings and a low consumption of sugars, fruits, dairy, animal fats, confectionaries and sugar-sweetened beverages [42]. Because the dietary intake of the Japanese population differs from that of Western populations, the intake and major sources of starch and sugars may differ in Japan compared with those in Western countries.

In the present study, we developed a comprehensive database on starch and seven types of sugars (sucrose, lactose, maltose, trehalose, glucose, fructose and galactose) and estimated starch and sugar intake and their food sources in Japan.

## 2. Materials and Methods

### 2.1. Development of a Food Composition Database

A comprehensive food composition database on starch and sugars was developed for food items included in STFCJ [40,41]. Information on starch and saccharides (glucose, fructose, galactose, sucrose, maltose, lactose and trehalose) was available for 880 of 2222 food items (39.6%) in STFCJ, with no information on total, free and naturally occurring sugar. Thus, we assigned the values of the remaining 1342 items that lacked information on starch and saccharides and estimated the values of total, free and naturally occurring sugar in all 2222 items. For 880 items with information on starch and saccharides, the values were based on analytical data (determined using enzymatic methods for starch or high-performance liquid chromatography for saccharides) for 396 items (45.0%), information on similar foods in STFCJ for 187 items (21.3%), recipes for 144 items (16.4%) and data from food composition databases of other countries (the US [43], the UK [44] and Australia [45]) for 153 items (17.4%).

#### 2.1.1. Total Sugar and Saccharides

**Figure 1:**
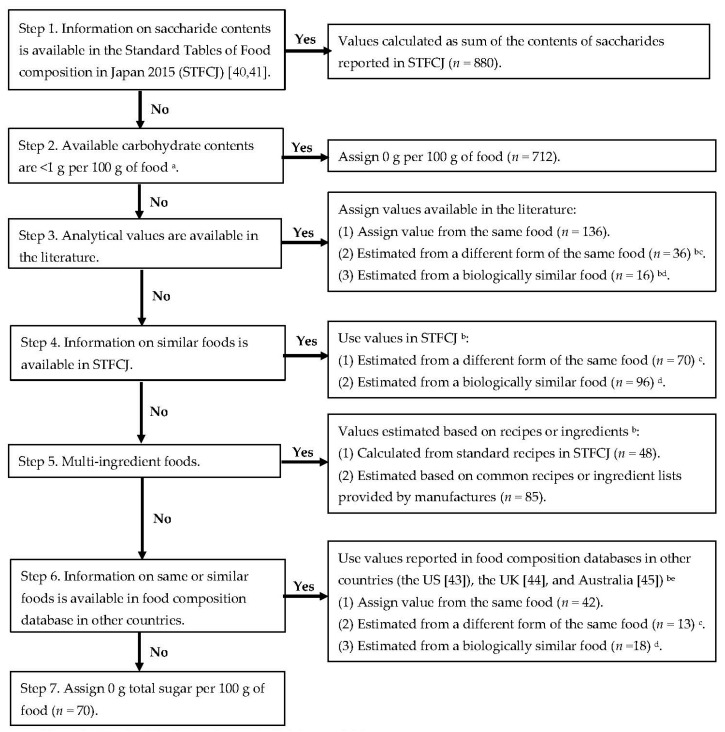
Flow chart for the development of total sugar database. ^a^ Available carbohydrate content was calculated as subtracting dietary fiber content (g/100 g of food) from carbohydrate that was calculated as subtracting the sum of contents (g/100 g of food) of water, protein, lipid and ash from 100 g of food in the Standard Tables of Food composition in Japan 2015 [40,41]. ^b^ Values were adjusted using the ratio of the dry weight between interested and referred food items. ^c^ This was applied to items such as cooked (i.e., boiled, salted, or dried) vegetables and seaweeds using the value of raw form. Total sugar content of the different form was calculated as follows: total sugar content of cooked food = total sugar content of raw food × dry weight of cooked food/dry weight of raw food (a unit of all values was g/100 g of food). ^d^ Estimation was performed in mainly cereals, vegetables, fruits and seaweeds belonging to same family or order. Values from a similar food item were calculated after comparing total energy and other nutrient contents as follows: total sugar content of food of interest = total sugar content of similar food × dry weight of interest food/ dry weight of similar food (a unit of all values was g/100 g of food). ^e^ When the value of a specific food item was available in several countries, the source of imports was considered primary and a similarity for total energy and other nutrient contents was a second consideration. When assigning data from the UK, disaccharide values were multiplied by 0.95 since values were shown as monosaccharide equivalents [44].

Total sugar was defined as the sum of all mono- and disaccharides, including glucose, fructose, galactose, sucrose, lactose, maltose and trehalose [46]. A database of total sugar and saccharides was developed according to the following seven-step method (Figure 1 and Table S1). For 880 items for which information on saccharide contents was available, the total sugar contents were calculated based on the values in the STFCJ [40,41] (Step 1). For the remaining 1342 items without saccharide content, a stepwise data-gathering strategy, proposed by Rand et al. [47], was used (Steps 2–7). For the step for assigning analytical values (Step 3), a literature search was conducted based on several databases, to identify studies that reported sugar content in Japanese foods. Search terms and the criteria of choosing values are shown in Table S1. Finally, the values from 12 papers [48,49,50,51,52,53,54,55,56,57,58,59] were used. For each food item, the mean or median value was used as a representative value.

#### 2.1.2. Starch

The starch content in each food was determined with a similar procedure to that used for sugars, although the procedure included a step that assigned a total sugar content subtracted from available carbohydrate content value (Step 7), followed by a step that assigned 0 g starch to the remaining foods (Step 8) (Table S2). For the step for assigning analytical values (Step 3), a literature search was conducted in a similar strategy to sugar to identify studies that reported starch contents in Japanese foods (Table S2). Finally, the values from two papers [60,61] were used. For each food item, the median value was used as a representative value.

#### 2.1.3. Free Sugar and Naturally Occurring Sugar

Free sugar was defined according to the WHO definition, as all mono- and disaccharides added to foods and beverages by the manufacturer, cook, or consumer and sugars naturally present in honey, syrups, fruit juices and fruit juices concentrate [62]. Free sugar contents in all food items were determined using a published stepwise method [63,64] based on the total sugar and saccharide contents as shown in Table S3. Contents of naturally occurring sugar were estimated by subtracting free sugar contents from total sugar contents.

### 2.2. Estimation of Starch and Sugar Intake

#### 2.2.1. Dietary Dataset

To estimate starch and sugar intake, the starch and sugar database was applied to dietary data obtained from a Japanese population with a wide age range: adults, schoolchildren, preschool children and toddlers. Table 1 shows the description of the dietary dataset that was used. For the adult population, we included 196 healthy men and 196 healthy women, aged 20–69 years, in 20 study areas in 47 prefectures, as described elsewhere [65,66]. On average, each study area included 20 participants, consisting of two men and two women in each of the five 10-year age groups (20–29, 30–39, 40–49, 50–59 and 60–69 years). For the schoolchild population, we included 389 third graders (aged 8–9 years) and 392 fifth graders (aged 10–11 years) from elementary schools and 409 the second graders (aged 13–14 years) from junior high school from 12 prefectures [67,68]. On an average, 90 children (30 children in each of third and fifth grades of elementary school and 30 children in the second grade of junior high school) were included from each prefecture. For the population of preschool children and toddlers, we included 380 preschool children aged 3–6 years and 373 toddlers aged 18–35 months from nursery facilities in 24 prefectures who were participants of the DONGuRI study (Dietary Observation and Nutrient intake for Good health Research In Japanese young children) [69]. Generally, 16 children (two boys and two girls aged 3, 4, 5 and 6 years) and 16 toddlers (four boys and four girls aged 18–23 months and 24–35 months) were included from each prefecture. For schoolchildren, preschool children and toddlers, all participants were recruited from schools and nursery facilities providing a uniformed lunch. Among all surveys, recruitment was conducted non-randomly to reach the planned number of participants in each sex and age (group) as mentioned above.

Detailed descriptions on dietary assessment procedures are described elsewhere [65,66,67,68,69]. Briefly, dietary information was collected using weighed dietary records (DRs) in all age groups. The number of recording days differed: non-consecutive four days for adults, non-consecutive three days for schoolchildren and preschool children and one day for toddlers. The dietary data included the weight of all food and beverages consumed by the participants and the amount of leftovers during the study period. For adults, all food and beverages consumed were weighed and recorded by participants. For schoolchildren, all food and beverages consumed as a uniformed lunch were weighed or recorded by dietitians of the schools or participants under the dietitian’s support. The food and beverages consumed except for a uniformed lunch were weighed and recorded by the guardians of participants. For toddlers and preschool children, all food and beverages consumed at nursery facilities were weighed and recorded by dietitians of the facilities, irrespective of the origin of food. The food and beverages consumed out of nursery facilities were weighed and recorded by the guardians of participants. Food codes of food and beverage items were assigned according to STFCJ [40,41]. The total number of items that appeared in the DR was 1376 for adults, 1175 for schoolchildren, 1172 for preschool children and 868 for toddlers. The intake of energy and available carbohydrate were calculated according to STFCJ [40,41].

All three surveys were conducted according to the guidelines in the Declaration of Helsinki and all procedures involving human subjects were approved by the Ethics Committee of the University of Tokyo, Faculty of Medicine (approval code No. 10005 for the survey in adults, No. 10653 for the survey in school children, and No. 10885 for the survey in preschool children and toddlers). Informed consent was obtained from all adult participants and all guardians of child participants.

#### 2.2.2. Statistical Analysis

The intake of energy, starch and sugars are presented as mean and standard deviation (SD) by age and sex groups. The habitual dietary intake was estimated based on the best-power method (performed with HabitDist [70]) to account for day-to-day variation [71,72], except for toddlers for whom only 1-day dietary data were available. The ratio of reported energy intake (EI) to estimated energy requirement (EER) was calculated in each participant using the sex-and age-specific EER for medium level of physical activity in Dietary Reference Intakes for Japanese, 2015 [73]. Physical activity level of EER was fixed to medium level for all participants due to the absence of accurate information on physical activity. The adherence to the recommended sugar intake advocated by WHO [62] (<10% and <5% of EI from free sugar) was calculated. Differences between sexes were analyzed using an independent *t*-test for mean intake and a chi-square test for the prevalence of excessive free sugar intake (≥10% or ≥5% of EI) [62]. The mean value of the contribution of each food group to starch and sugar intake was calculated for both sexes combined because separate analyses for male and female subjects showed similar food group contribution patterns (data not shown). Food groups were defined based on culinary usage and nutrient profile similarities, mainly according to STFCJ [40,41]. All statistical analyses were performed using SAS version 9.4 (Institute Inc., Cary, North Carolina, USA). All reported *P* values are two-sided and those <0.05 were considered statistically significant.

## 3. Results

The present analysis included the participants who completed both anthropometric measurements and DR: 368 toddlers, 376 preschool children, 915 schoolchildren and 392 adults (Table S4). The mean EI/EER ranged from 0.92 to 1.05 for all age groups except for toddlers (1.22 for boys and 1.23 for girls).

The estimated intake in each sex and age group is shown in Table 2 (for available carbohydrate, starch, total sugar, sucrose, fructose, naturally occurring sugar and free sugar) and Table S5 (for energy and other sugars). The absolute mean intake ranged from 55.6 g/day (female toddlers) to 206.0 g/day (male schoolchildren) for starch and from 46.1 g/day (female toddlers) to 68.7 g/day (male schoolchildren) for total sugar. The energy-adjusted mean intake ranged from 32.5% of energy (%E) (female toddlers) to 36.6 %E (male schoolchildren) for starch and from 10.7 %E (male adults) to 17.5 %E (female toddlers) for total sugar. Among all age and sex groups, the top total sugar contributor was sucrose, followed by, glucose, lactose and fructose, with small contributions from maltose, trehalose and galactose. While the absolute amount of naturally occurring sugar consumed was on average similar to that of free sugar in preschool children and schoolchildren, the former was higher in toddlers and the latter was higher in adults. Concerning adherence to WHO recommendations for free sugar, almost one fifth of toddlers consumed ≥10 %E and those who consumed ≥5 %E were >50%. The prevalence of excessive free sugar intake in other age groups ranged from 3.1% to 13.3% (for ≥10 %E) and from 56.1% to 92.1% (for ≥5 %E). There were no sex differences in starch and sugar intake among toddlers except for absolute starch intake. The absolute mean intakes of starch and several sugars were higher in male subjects than in female subjects among preschool children and schoolchildren. For adults, the intake of naturally occurring sugar was higher in women. On the other hand, energy-adjusted mean intake (%E) of starch was higher in male subjects for schoolchildren and adults, while the sugar values were higher in female subjects, (except for free sugar in schoolchildren). The prevalence of participants consuming ≥5 %E from free sugar was higher in female subjects for schoolchildren and adults.

The major food sources are shown in Table 3 (for available carbohydrate, starch, total sugar, sucrose, fructose, naturally occurring sugar and free sugar) and Table S6 (for other sugars). Rice and grains contributed to 39.3–46.4% of available carbohydrate and 58.8–65.4% of starch, depending on age. While dairy products, fruits, vegetables, seasonings and bread were major contributor to naturally occurring sugar (76.4–87.4%), free sugar was mainly derived from confectionaries, sugars and jams, sugar-sweetened beverages and seasonings (82.3–84.3%). These foods were thus major sources of total sugar (83.8–90.3%). For each of sugars, the major contributors varied but a considerably large amount of sugars consumed was derived from only a few foods (e.g., confectionaries, fruits, sugars and jams, sugar-sweetened beverages and vegetables for sucrose (73.0–85.5%) and fruits, vegetables, sugar-sweetened beverages, bread, fruit juices and seasonings for fructose (86.9–90.8%)). The contribution of fruit juices to sugars was relatively small: 2.7–7.7% for fructose; 2.2–6.3% for free sugar; and 1.3–3.5% for total sugar.

## 4. Discussion

To our knowledge, this is the first study to provide a comprehensive description of the estimated intakes and sources of starch and sugar subtypes in a non-Western country. According to previous Western studies [27,28,29,30,31], the mean absolute intake of starch was comparable to that in the present study. However, the major food source in Western surveys differed from the present study. In a survey conducted in 10 European countries [27], bread was the top contributor of starch intake among all countries. In contrast, the top contributor was rice and grains in the present Japanese population, with a relatively high consumption (mean: 171–356 g/day).

For total sugar, the mean absolute intake of the present Japanese toddlers (aged ≤ 2 years) was comparable to that of Finnish breast-fed or Icelandic infants (both aged ≤ 1 year) and was lower than that of toddlers aged ≤3 years in other Western countries [28,32]. For preschool children (aged 3–6 years) in the present study, the value was lower compared with that of Western children aged 4–10 years [28,32]. Similarly, the value of schoolchildren (aged 8–14 years) in the present study was lower than that of Western children aged 9–18 years [28,30,32]. The value for present Japanese adults was comparable to that of Italian adults [34] and was lower than that of other Western adults [27,28,30,32,33,74]. One possible reason for a relatively lower total sugar intake in the present study could be due to the low consumption of sugar-sweetened beverages (mean: 43–112 g/day) compared with Western countries (mean: 120–570 mL/day for adults [75] and 66–732 mL/day in children [76]). These differences may also cause the lower contribution of sugar-sweetened beverages to total sugar in the present study than most of Western countries [28,74,77,78]. In contrast, sugars and jams and confectionaries were the major sources in both the present and Western studies [27,28,74,77,78,79,80], despite relatively low mean consumptions (mean: 3–9 g/day for sugars and jams and 43–69 g/day for confectionaries). Interestingly, unlike most of Western countries [27,28,77,78,79,80], vegetables was one of the major contributors in the present study. This may be due to a high vegetable consumption (mean: 121–239 g/day) in combination with relatively low sugar-sweetened beverage intake.

For sucrose, the mean absolute intake of our Japanese toddlers was higher than that of Finnish infants aged 1 year and lower than that of Finnish toddlers aged 2–3 years [32]. Similarly to total sugar, our preschool children, schoolchildren and adults had lower intake compared with Western children (aged 4–6 years and aged 7–14 years [32]) and adults [32,33] (except for Italian adults with comparable intake [34]).

For other saccharides, a few countries reported absolute intakes within a narrower age range, compared with total sugar and sucrose. The absolute amount of fructose for schoolchildren and adults in this Japanese population was lower compared with that of Western schoolchildren (aged 7–18 years) [35] and adults [33,34,35]. The lower sugar-sweetened-beverage intake in this Japanese population mentioned above could also explain lower fructose intake (as well as sucrose) and may be a cause of the low contribution to fructose. Additionally, the absolute intake of glucose in our adults was lower than that of Western counterparts [33], while that of lactose [33,34] and maltose [33] was comparable.

Concerning adherence to WHO recommendations for free sugar [62], the prevalence of ≥10 %E in this Japanese population was lower than Western countries, irrespective of sexes and age groups [29,78,80,81]. The prevalence of ≥5 %E in this Japanese population was also lower, except for female adults with the comparable prevalence to Western adults [78,80,81]. Therefore, the priority of free sugar reduction in this Japanese population may relatively be lower than that in Western countries.

There are several limitations to the present study. First, our participants were not representative samples but volunteers and were possibly health conscious. Particularly, adult participants (except for those aged 60 years and above) were workers in welfare facilities. For toddlers and preschool children, participants were recruited from nursery facilities, while only 45% of the children aged 1–6 years attended nursery school in Japan [82]. Thus, the present results may not be directly applicable to the general Japanese population. Nevertheless, it should be noted that the mean height and weight values of adult and child participants by sex and age were comparable to those of the general Japanese population [42,83,84].

Second, we could not estimate habitual intake in toddlers by accounting for day-to-day variation because we only had 1-d DR data. Therefore, the results of this age group, especially those of the prevalence of excessive free sugar intake, should be interpreted with caution, although fewer days were needed to assess habitual dietary intake in younger children compared with older children and adults [85]. However, we mainly discussed the mean value of each age group; and thus, the influence of this matter should be small. Moreover, there is a possibility of seasonal variation in dietary intake because the surveys were conducted in a limited period. This might be a cause of bias into the assessment of average dietary patterns over the year, considering seasonal differences in food intake among Japanese [86,87].

Third, while DRs can obtain detailed information on individual diet, this method is based on a self-report and the possibility of measurement error remains. Nevertheless, measurement errors should be attenuated when the energy adjustment was conducted [88]. In the present study, the mean EI/EER was almost 1.00 for all age groups except for toddlers (1.22 for boys and 1.23 for girls). Therefore, if a degree and a direction of reporting errors in starch and sugar intakes were similar to those of EI, the reported absolute and energy-adjusted values in this Japanese population are likely accurate, at least at the group level, with the exception of toddlers. For toddlers, although the intakes seem over-estimated, the intakes (especially for total sugar) were still relatively low; and thus, this matter may have a small impact on the results of this population.

Finally, there were unavoidable limitations in dataset development. Particularly, the starch contents in other items were determined using the values of similar food items (*n* = 282, recipes (*n* = 161), or the difference between total sugar and available carbohydrate contents (*n* = 100). These procedures may lead to the under- or over-estimation of starch intake. because we could not consider the difference in food items among biologically similar foods, the changes that occur during cooking and processing [64] and the oligosaccharide and sugar alcohol contents. The same is true for sugar intake derived from items whose values were determined from the values of similar food items (*n* = 239) or recipes (*n* = 133). However, the measurement error caused by the remaining 1592 food items, which represented about 89% (for adults) to 94% (for toddlers) of available carbohydrate intake, should be minimal, because the starch and sugar contents for these were determined using saccharide (*n* = 880) or available carbohydrate (*n* = 712) contents in the STFCJ [40,41]. In any case, further research on starch and sugar content in food items without values in the food composition table in Japan is needed to accurately estimate the starch and sugar intake in the current Japanese population.

## 5. Conclusions

This study provided a comprehensive picture of starch and sugar intake and their food sources in Japan. The mean starch intake in this Japanese population was comparable to that in Western countries, while this Japanese population had a relatively low total sugar intake and the major contributors somewhat differed from those of Western countries. These differences could be explained by the difference between Japanese and Western diets. The database developed in this study and the present findings provide a foundation for future research.

## Figures and Tables

**Table 1:** Description of dietary dataset.

	Toddlers ^a^	Preschool Children ^a^	Schoolchildren ^b^	Adults ^c^
Data collection period	October to December 2015	October to December 2015	November to December 2014	February to March 2013
**Population characteristics**				
Number of participants	373	380	1190 ^d^	392
Age	18–35 months	3–6 years	8–14 years	20–69 years
Study setting	Nursery facilities (*n* = 315)	Nursery facilities (*n* = 315)	Elementary schools (*n* = 14) and junior high schools (*n* = 13)	199 welfare facilities (for aged <60 years) and neighbors or acquaintances of the dietitians of welfare facilities (aged ≥60 years)
Study area ^e^	24 of 47 prefectures	24 prefectures	12 prefectures	20 study areas ^f^
Recruitment	Four boys and four girls aged 18–23 months and 24–35 months (16 children in total)	Two boys and two girls aged 3, 4, 5 and 6 years (16 children in total)	30 children in each of third and fifth grades of elementary school and 30 children in the second grade of junior high school (90 children in total)	Two men and two women from each of five 10-year age groups (20–29, 30–39, 40–49, 50–59 and 60–69 years, 20 participants in total)
Exclusion criteria	Under diet therapy by a doctor or a dietitian at the time of the study; having particular dietary habits (such as vegetarian); or having guardians (mothers in almost all cases) whose occupation was dietitians or medical doctors	Under diet therapy by a doctor or a dietitian at the time of the study; having particular dietary habits (such as vegetarian); or having guardians (mothers in almost all cases) whose occupation was dietitians or medical doctors	None	Dietitian; not living in the prefecture in which the facility was located or its adjacent prefectures; under diet therapy by a doctor or a dietitian at the time of the study or within one year before the study; pregnant or lactating women; or history of educational admission for diabetes mellitus
Characteristics of DR *				
Number of days	1 day: one weekday with a uniformed lunch provided by facilities	3 days: two weekdays with a uniformed lunch provided by facilities and one weekend day without a uniformed lunch provided by facilities	3 days: two weekdays with a uniformed lunch provided by schools and one weekend day without a uniformed lunch provided by schools	4 days: three weekdays and one weekend day

* DR, dietary record. ^a^ More details described elsewhere [69]. ^b^ More details described elsewhere [67,68]. ^c^ More details described elsewhere [65,66]. ^d^ Consisting of 389 third and 392 fifth graders from elementary schools and 409 the second graders from junior high schools. ^e^ Selected in consideration of geographical location (e.g., north or south, rural or urban) and study feasibility. ^f^ Consisting of 23 prefectures, three study areas included two adjacent prefectures.

**Table 2:** Estimated intakes of available carbohydrate, starch, total sugar, sucrose, fructose, naturally occurring sugar and free sugar in Japanese children and adults.

	Toddlers (Aged 18–35 Months) ^a^				Preschool Children (Aged 3–6 Years) ^b^				Schoolchildren (Aged 8–14 Years) ^b^				Adults (Aged 20-69 Years) ^b^			
	Boys (*n* = 183)		Girls (*n* = 185) ^c^		Boys (*n* = 186)		Girls (*n* = 190) ^c^		Boys (*n* = 435)		Girls (*n* = 480) ^c^		Men (*n* = 196)		Women (*n* = 196) ^c^	
	Mean	SD	Mean	SD	Mean	SD	Mean	SD	Mean	SD	Mean	SD	Mean	SD	Mean	SD
**Absolute Value (g/day)**																
Available carbohydrate	151.7	28.9	141.9 **	29.2	188.1	28.0	173.9 ***	25.8	295.2	63.2	258.2 ***	39.7	293.3	62.5	238.8 ***	43.2
Starch	61.1	22.5	55.6 *	19.8	116.4	21.1	107.8 ***	19.7	206.0	50.4	176.3 ***	30.3	203.4	48.3	153.8 ***	31.5
Total sugar	47.5	16.4	46.1	16.9	57.5	12.6	53.0 ***	10.6	68.7	19.7	64.1 ***	15.8	62.2	24.0	64.5	20.4
Sucrose	19.5	9.6	18.2	9.0	25.0	6.5	22.9 ***	4.9	29.3	10.8	27.7	8.4	31.5	15.6	34.0	12.9
Fructose	7.8	4.3	7.6	5.4	10.0	3.7	9.3	2.7	11.1	3.8	10.3 **	4.0	10.1	4.3	9.9	4.0
Naturally occurring Sugar	30.1	11.9	27.9	9.8	30.0	5.8	27.3 ***	5.5	35.9	10.1	33.8 ***	8.1	26.5	11.5	28.8 *	11.3
Free sugar	17.4	11.9	18.2	12.7	27.4	9.3	25.6 *	7.1	32.8	13.6	30.3 **	10.3	35.7	19.0	35.7	13.7
**Energy-Adjusted Value (% of Energy)**																
Available carbohydrate	54.2	6.4	54.4	6.4	53.0	2.9	52.9	2.8	52.5	3.2	51.7 ***	3.1	50.5	6.3	51.0	3.8
Starch	32.9	6.9	32.5	6.4	32.8	3.7	32.8	3.0	36.6	4.2	35.3 ***	3.4	35.1	5.6	33.0 ***	4.6
Total sugar	17.0	5.3	17.5	5.2	16.2	2.9	16.1	2.8	12.3	2.9	12.8 **	2.6	10.7	4.0	13.6 ***	3.4
Sucrose	6.9	3.2	6.9	3.2	7.0	1.6	6.9	1.2	5.2	1.6	5.5 **	1.4	5.4	2.4	7.1 ***	2.2
Fructose	2.8	1.5	2.9	1.8	2.8	1.0	2.9	0.8	2.0	0.6	2.1 *	0.7	1.8	0.8	2.1 ***	0.8
Naturally occurring sugar	10.8	4.3	10.7	3.4	8.5	1.7	8.4	1.6	6.4	1.5	6.8 ***	1.3	4.6	1.8	6.1 ***	2.1
Free sugar	6.1	4.0	6.9	4.4	7.6	2.2	7.7	1.9	5.8	2.1	6.0	1.9	6.1	3.2	7.4 ***	2.4
≥10% of energy intake from free sugar (%)	15.3		21.1		12.9		11.6		4.1		3.1		8.7		13.3	
≥5% of energy intake from free sugar (%)	51.4		59.5		90.9		92.1		61.6		68.8*		55.6		87.8 ***	

SD, standard deviation. ^a^ Estimated using 1-day dietary data. ^b^ Habitual intake was calculated based on the best-power method (performed with HabitDist [70]) by using 3-day dietary data for preschool children and schoolchildren or 4-day dietary data for adults [71,72]. ^c^ Significantly different from the corresponding male group determined by independent *t*-test for continuous variables and chi-square test for categorical variables; * *p* < 0.05, ** *p* < 0.01, *** *p* < 0.001.

**Table 3:** Contribution (%) of each food group to intakes of available carbohydrate, starch, total sugar, sucrose, fructose, naturally occurring sugar and free sugar in Japanese children and adults ^ab.^

Nutrients	Food Groups ^cd^	Toddlers (Aged 18–35 Months) (*n* = 368)	Preschool Children (Aged 3–6 Years) (*n* = 376)	Schoolchildren (Aged 8–14 Years) (*n* = 915)	Adults (Aged 20–69 Years) (*n* = 392)
Available carbohydrate	Rice and grains	42.5	39.3	46.4	44.5
	Confectionaries	11.7	13.5	10.0	9.7
	Fruits	8.4	6.3	3.2	3.2
	Dairy products	8.0	5.1	4.5	1.7
	Bread	4.7	7.1	8.1	6.9
	Noodle	2.6	5.2	6.6	8.1
	Others	22.1	23.4	21.1	25.8
Starch	Rice and grains	65.4	58.8	62.3	61.2
	Confectionaries	11.3	11.3	7.7	7.8
	Bread	6.9	10.0	10.6	9.2
	Noodle	4.1	7.7	8.8	11.4
	Others	12.4	12.3	10.6	10.4
Total sugar	Dairy products	22.7	15.6	17.5	6.1
	Fruits	20.9	16.9	10.0	10.3
	Confectionaries	14.5	19.6	17.6	17.5
	Vegetables	10.8	9.7	13.1	14.3
	Sugar-sweetened beverages ^e^	8.9	11.0	9.4	11.5
	Sugars and jams	6.9	7.1	9.2	13.2
	Seasonings	5.6	6.4	9.0	10.9
	Others	9.7	13.6	14.1	16.2
Sucrose	Confectionaries	27.3	32.5	29.0	26.2
	Fruits	24.9	18.0	12.0	11.2
	Sugars and jams	16.3	15.5	21.5	24.0
	Sugar-sweetened beverages ^e^	9.3	10.3	8.7	11.6
	Vegetables	7.7	6.3	7.6	6.6
	Others	14.5	17.4	21.2	20.4
Fructose	Fruits	39.3	30.8	18.6	17.1
	Vegetables	27.5	24.6	37.9	37.7
	Sugar-sweetened beverages ^e^	9.9	13.5	12.2	10.7
	Bread	5.4	7.7	10.5	10.7
	Fruit juices	5.1	7.7	3.5	2.7
	Seasonings	3.5	5.1	7.0	8.0
	Others	9.2	10.7	10.3	13.1
Naturally occurring sugar	Dairy products	33.5	25.9	27.9	10.9
	Fruits	30.6	28.0	16.1	17.8
	Vegetables	16.8	17.9	23.8	29.8
	Seasonings	3.4	4.7	7.6	9.3
	Bread	3.0	5.9	7.5	8.6
	Others	12.6	17.7	17.1	23.6
Free sugar	Confectionaries	33.5	34.4	30.3	26.2
	Sugars and jams	22.0	17.9	23.6	26.5
	Sugar-sweetened beverages ^e^	16.3	18.4	14.7	15.6
	Seasonings	12.5	11.6	14.3	15.9
	Others	15.7	17.7	17.1	15.9

^a^ Values are means. The mean value of contribution was calculated by using 1-day dietary data (for toddlers) or the mean of 3- or 4-day dietary data (for other age groups). ^b^ Food groups with <7% contribution in all populations are not shown and combined into others. ^c^ Twenty-four food groups were defined based on the culinary usage and the similarity of nutrient profiles of the foods, mainly according to the Standard Tables of Food composition in Japan 2015 [40,41]. They consisted of other grain products, potatoes, pulses and nuts, mushrooms, seaweeds, fish and shellfish, meats, eggs, fat and oil, alcoholic beverages, vegetable juices, tea and coffee and other foods in addition to the items listed in this table. ^d^ Food groups are listed in descending order of the contribution in toddlers. ^e^ Consisting of soda, sports drinks, fruit drinks, milk beverages and pre-sweetened tea and coffee.

## Supplementary Materials

The following are available online at http://www.mdpi.com/2072-6643/10/10/1474/s1, Table S1: Number of food items in each step in the development of total sugar database [40,41,43,44,45,48,49,50,51,52,53,54,55,56,57,58,59,89,90,91,92,93,94], Table S2: Number of food items in each step in the development of starch database [40,41,43,44,45,58,60,61,95], Table S3: Number of food items in each step in the development of free sugar database [40,41,43,96], Table S4: Basic characteristic of Japanese children and adults [70,71,72,73], Table S5: Estimated intakes of energy, maltose, lactose, trehalose, glucose and galactose in Japanese children and adults [70,71,72], Table S6: Contribution (%) of each food group to intakes of maltose, lactose, trehalose, glucose and galactose in Japanese children and adults [40,41].

## References

[1] Buyken A.E., Mela D.J., Dussort P., Johnson I.T., Macdonald I.A., Stowell J.D., Brouns F.J.P.H. (2018). Dietary carbohydrates: A review of international recommendations and the methods used to derive them. Eur. J. Clin. Nutr..

[2] Erickson J., Sadeghirad B., Lytvyn L., Slavin J., Johnston B.C. (2017). The Scientific Basis of Guideline Recommendations on Sugar Intake: A Systematic Review. Ann. Intern. Med..

[3] Moynihan P.J., Kelly S.A.M. (2014). Effect on Caries of Restricting Sugars Intake: Systematic Review to Inform WHO Guidelines. J. Dent. Res..

[4] Te Morenga L., Mallard S., Mann J. (2012). Dietary sugars and body weight: Systematic review and meta-analyses of randomised controlled trials and cohort studies. BMJ.

[5] Imamura F., O’Connor L., Ye Z., Mursu J., Hayashino Y., Bhupathiraju S.N., Forouhi N.G. (2015). Consumption of sugar sweetened beverages, artificially sweetened beverages, and fruit juice and incidence of type 2 diabetes: Systematic review, meta-analysis, and estimation of population attributable fraction. BMJ.

[6] Te Morenga L.A., Howatson A.J., Jones R.M., Mann J. (2014). Dietary sugars and cardiometabolic risk: Systematic review and meta-analyses of randomized controlled trials of the effects on blood pressure and lipids. Am. J. Clin. Nutr..

[7] Yang Q., Zhang Z., Gregg E.W., Flanders W.D., Merritt R., Hu F.B. (2014). Added Sugar Intake and Cardiovascular Diseases Mortality Among US Adults. JAMA Intern. Med..

[8] Hauner H., Bechthold A., Boeing H., Brönstrup A., Buyken A., Leschik-Bonnet E., Linseisen J., Schulze M., Strohm D., Wolfram G. (2012). Evidence-based guideline of the German nutrition society: Carbohydrate intake and prevention of nutrition-related diseases. Ann. Nutr. Metab..

[9] Gibson S., Gunn P., Wittekind A., Cottrell R. (2013). The Effects of Sucrose on Metabolic Health: A Systematic Review of Human Intervention Studies in Healthy Adults. Crit. Rev. Food Sci. Nutr..

[10] Touger-Decker R., Van Loveren C. (2003). Sugars and dental caries. Am. J. Clin. Nutr..

[11] Lingström P., Van Houte J., Kashket S. (2000). Food starches and dental caries. Crit. Rev. Oral Biol. Med..

[12] Sluijs I., van der Schouw Y.T., van der A D.L., Spijkerman A.M., Hu F.B., Grobbee D.E., Beulens J.W. (2010). Carbohydrate quantity and quality and risk of type 2 diabetes in the European Prospective Investigation into Cancer and Nutrition-Nethelands (EPIC-NL) study. Am. J. Clin. Nutr..

[13] Hodge A.M. (2004). Glycemic index and dietary fiber and the risk of type 2 diabetes. Diabetes Care.

[14] Barclay A., Flood V., Rochtchina E., Mitchell P., Brand-Miller J. (2007). Glycemic Index, Dietary Fiber, and Risk of Type 2 Diabetes in a Cohort of Older Australians. Diabetes Care.

[15] Meyer K.A., Kushi L.H., Jacobs D.R., Slavin J., Sellers T.A., Folsom A.R. (2000). Carbohydrates, dietary fiber, and incident type 2 diabetes in older women. Am. J. Clin. Nutr..

[16] Sluijs I., Beulens J.W.J., van der Schouw Y.T., van der A D.L., Buckland G., Kuijsten A., Schulze M.B., Amiano P., Ardanaz E., Balkau B. (2013). Dietary Glycemic Index, Glycemic Load, and Digestible Carbohydrate Intake Are Not Associated with Risk of Type 2 Diabetes in Eight European Countries.

[17] Montonen J., Järvinen R., Knekt P., Heliövaara M., Reunanen A. (2007). Consumption of sweetened beverages and intakes of fructose and glucose predict type 2 diabetes occurrence. J. Nutr..

[18] Ahmadi-Abhari S., Luben R.N., Powell N., Bhaniani A., Chowdhury R., Wareham N.J., Forouhi N.G., Khaw K.T. (2014). Dietary intake of carbohydrates and risk of type 2 diabetes: The European Prospective Investigation into Cancer-Norfolk study. Br. J. Nutr..

[19] Tsilas C.S., de Souza R.J., Mejia S.B., Mirrahimi A., Cozma A.I., Jayalath V.H., Ha V., Tawfik R., Di Buono M., Jenkins A.L. (2017). Relation of total sugars, fructose and sucrose with incident type 2 diabetes: A systematic review and meta-analysis of prospective cohort studies. Can. Med. Assoc. J..

[20] Alhazmi A., Stojanovski E., McEvoy M., Garg M.L. (2012). Macronutrient intakes and development of type 2 diabetes: A systematic review and meta-analysis of cohort studies. J. Am. Coll. Nutr..

[21] Janket S.-J., Manson J.E., Sesso H., Buring J.E., Liu S. (2003). A Prospective Study of Sugar Intake and Risk of Type 2 Diabetes in Women. Diabetes Care.

[22] Schulze M.B., Schulz M., Heidemann C., Schienkiewitz A., Hoffmann K., Boeing H. (2008). Carbohydrate intake and incidence of type 2 diabetes in the European Prospective Investigation into Cancer and Nutrition (EPIC)-Potsdam Study. Br. J. Nutr..

[23] Ter Horst K.W., Serlie M.J. (2017). Fructose consumption, lipogenesis, and non-alcoholic fatty liver disease. Nutrients.

[24] Tappy L. (2018). Fructose metabolism and noncommunicable diseases: Recent findings and new research perspectives. Curr. Opin. Clin. Nutr. Metab. Care.

[25] Chiu S., Sievenpiper J.L., De Souza R.J., Cozma A.I., Mirrahimi A., Carleton A.J., Ha V., Di Buono M., Jenkins A.L., Leiter L.A. (2014). Effect of fructose on markers of non-alcoholic fatty liver disease (NAFLD): A systematic review and meta-analysis of controlled feeding trials. Eur. J. Clin. Nutr..

[26] David Wang D., Sievenpiper J.L., De Souza R.J., Cozma A.I., Chiavaroli L., Ha V., Mirrahimi A., Carleton A.J., Di Buono M., Jenkins A.L. (2014). Effect of fructose on postprandial triglycerides: A systematic review and meta-analysis of controlled feeding trials. Atherosclerosis.

[27] Cust A.E., Skilton M.R., van Bakel M.M.E., Halkjaer J., Olsen A., Agnoli C., Psaltopoulou T., Buurma E., Sonestedt E., Chirlaque M.D. (2009). Total dietary carbohydrate, sugar, starch and fibre intakes in the European Prospective Investigation into Cancer and Nutrition. Eur. J. Clin. Nutr..

[28] Public Health England, The Food Standards Agency (2014). National Diet and Nutrition Survey Results from Years 1, 2, 3 and 4 (Combined) of the Rolling Programme (2008/2009–2011/2012).

[29] Lluch A., Maillot M., Gazan R., Vieux F., Delaere F., Vaudaine S., Darmon N. (2017). Individual Diet Modeling Shows How to Balance the Diet of French Adults with or without Excessive Free Sugar Intakes. Nutrients.

[30] Ruiz E., Rodriguez P., Valero T., Ávila J.M., Aranceta-Bartrina J., Gil Á., González-Gross M., Ortega R.M., Serra-Majem L., Varela-Moreiras G. (2017). Dietary intake of individual (Free and intrinsic) sugars and food sources in the Spanish population: Findings from the ANIBES study. Nutrients.

[31] Sette S., Le Donne C., Piccinelli R., Arcella D., Turrini A., Leclercq C. (2011). The third Italian National Food Consumption Survey, INRAN-SCAI 2005-06—Part 1: Nutrient intakes in Italy. Nutr. Metab. Cardiovasc. Dis..

[32] Newens K.J., Walton J. (2016). A review of sugar consumption from nationally representative dietary surveys across the world. J. Hum. Nutr. Diet..

[33] Linseisen J., Schulze M.B., Saadatian-Elahi M., Kroke A., Miller A.B., Boeing H. (2003). Quantity and quality of dietary fat, carbohydrate, and fiber intake in the German EPIC cohorts. Ann. Nutr. Metab..

[34] Marangoni F., Brignoli O., Cricelli C., Poli A. (2017). Lifestyle and specific dietary habits in the Italian population: Focus on sugar intake and association with anthropometric parameters—The LIZ (Liquidi e Zuccheri nella popolazione Italiana) study. Eur. J. Nutr..

[35] Sluik D., Engelen A.I., Feskens E.J. (2015). Fructose consumption in the Netherlands: The Dutch national food consumption survey 2007–2010. Eur. J. Clin. Nutr..

[36] Marriott B.P., Cole N., Lee E. (2009). National Estimates of Dietary Fructose Intake Increased from 1977 to 2004 in the United States. J. Nutr..

[37] Zhou B.F., Stamler J., Dennis B., Moag-Stahlberg A., Okuda N., Robertson C., Zhao L., Chan Q., Elliott P. (2003). Nutrient intakes of middle-aged men and women in China, Japan, United Kingdom, and United States in the late 1990s: The INTERMAP Study. J. Hum. Hypertens..

[38] Wang Z., Uchida K., Ohnaka K., Morita M., Toyomura K., Kono S., Ueki T., Tanaka M., Kakeji Y., Maehara Y. (2014). Sugars, sucrose and colorectal cancer risk: The Fukuoka colorectal cancer study. Scand. J. Gastroenterol..

[39] Saido M., Asakura K., Masayasu S., Sasaki S. (2016). Relationship Between Dietary Sugar Intake and Dental Caries Among Japanese Preschool Children with Relatively Low Sugar Intake (Japan Nursery School SHOKUIKU Study): A Nationwide Cross-Sectional Study. Matern. Child Health J..

[40] Science and Technology Agency (2015). Standard Tables of Food Composition in Japan-2015-(Seventh Revised Edition).

[41] Science and Technology Agency (2016). Standard Tables of Food Composition in Japan-2015-(Seventh Revised Edition) Addendum-2016.

[42] Ministry of Health, Labour and Welfare of Japan (2013). The National Health and Nutrition Survey in Japan. http://www.mhlw.go.jp/bunya/kenkou/eiyou/h25-houkoku.html.

[43] US Department of Agriculture (2015). Composition of Foods Raw, Processed, Prepared USDA National Nutrient Database for Standard Reference, Release 28. http://www.ars.usda.gov/ba/bhnrc/ndl.

[44] Public Health England McCance and Widdowson’s The Composition of Foods integrated dataset (CoFIDS). https://www.gov.uk/government/publications/composition-of-foods-integrated-dataset-cofid.

[45] Food Standards Australia New Zealand NUTTAB 2010 Food Composition Database. http://www.foodstandards.gov.au/science/monitoringnutrients/nutrientables/Pages/default.aspx.

[46] Food and Agriculture Organization of the United Nations Carbohydrates in Human Nutrition: Report of a Joint FAO/WHO Expert Consultation: Food and Agriculture Organization. http://www.fao.org/docrep/W8079E/W8079E00.htm.

[47] Rand W.M., Pennington J.A.T., Murphy P., Klensin J.C. (1991). Compiling Data for Food Composition Data Bases.

[48] Yamazawa K. (1982). The Free Sugars of Arrowheads. Bull. Tokai Women’s Jr. Coll..

[49] Ishiwata H., Yano S., Okuda S., Kotani T., Tsuji K. (1988). Analysis of contents of fatty acids, sterols, saccharides and dietary fibers in confectioneries on the market. Nippon Eiyo Shokuryo Gakkaishi.

[50] Yamamoto S., Taniguchi H., Sarukura N., Tsao H., Tseng A., Takeichi H. (2009). Development of a Food Composition Database of Monosaccharides and Disaccharides in Sweet Snacks and Beverages. J. Jpn. Diet. Assoc..

[51] Takeichi H., Wakikawa N., Taniguchi H., Sarukura N., Tsao H., Tseng A., Yamamoto S. (2010). Concentrations of Monosaccharides and Disaccharides in Commercial Sweet Snacks. J. Jpn. Diet. Assoc..

[52] Yoshikawa K., Murata Y., Murao R., Inagaki K., Terashita T., Shishiyama J. (1993). Preparation of Tables of Sugars in Food and Survey of Daily Sugar Intake. Food Hyg. Saf. Sci..

[53] Masuda T., Kawano A., Kitahara K., Nagashima K., Aikawa Y., Arai S. (2003). Quantitative determination of sugars and myo-inositol in citrus fruits grown in Japan using high-performance anion-exchange chromatography. J. Nutr. Sci. Vitaminol. (Tokyo).

[54] Yu X., Xu B., Sawamura M. (2004). Determination of Sugar and Organic Acid Contents in Yuzu Juices from Different Districts of Japan by HPLC. J. Jpn. Soc. Hortic. Sci..

[55] Tanaka H., Date C., Okazaki K., Yoshikawa K., Baba A., Hayashi M., Tanaka Y., Ishii R., Shoji H., Owada K. (1983). The contents of sucrose, fructose, glucose, maltose, lactose and sorbitol in daily foods -sugar composition of daily foods-. Jpn. J. Public Health.

[56] Ishii Y. (1983). Sugar Components of Some Dry Fruits. Nippon Eiyo Shokuryo Gakkaishi.

[57] Oku K., Sawatani I., Chaen H., Fukuda S., Kurimoto M. (1998). Trehalose Content in Foods. Nippon Shokuhin Kagaku Kogaku Kaishi.

[58] Yoshida H., Sugahara T., Hayashi J. (1982). Studies on Free Sugars, Free Sugar alcohols and Organic Acids of Edible Mushrooms. Nippon Shokuhin Kogyo Gakkaishi.

[59] Wu M.C., Chen C.S. (1998). Variation of Sugars Distribution in Various Parts of Pitaya (*Hylocereus undatus Britt.* et Rose). Food Preserv. Sci..

[60] Hase S., Yasui T. (1980). Studies on Determination of Starch in Agricultural Products Part 2 Determination of starch in pulse and its processed products. Rep. Natl. Food Res. Inst..

[61] Wamg P.S., Igarashi O., Fujimaki M. (1964). Determination of Starch in Meat Products. Nippon Shokuhin Kogyo Gakkaish.

[62] World Health Organization (2015). Guideline: Sugars Intake for Adults and Children.

[63] Mok A., Ahmad R., Rangan A., Louie J.C.Y. (2018). Intake of free sugars and micronutrient dilution in Australian adults. Am. J. Clin. Nutr..

[64] Louie J.C.Y., Moshtaghian H., Boylan S., Flood V.M., Rangan A.M., Barclay A.W., Brand-Miller J.C., Gill T.P. (2015). A systematic methodology to estimate added sugar content of foods. Eur. J. Clin. Nutr..

[65] Asakura K., Uechi K., Sasaki Y., Masayasu S., Sasaki S. (2014). Estimation of sodium and potassium intakes assessed by two 24 h urine collections in healthy Japanese adults: A nationwide study. Br. J. Nutr..

[66] Asakura K., Uechi K., Masayasu S., Sasaki S. (2016). Sodium sources in the Japanese diet: Difference between generations and sexes. Public Health Nutr..

[67] Asakura K., Sasaki S. (2017). School lunches in Japan: Their contribution to healthier nutrient intake among elementary-school and junior high-school children. Public Health Nutr..

[68] Asakura K., Sasaki S. (2017). SFA intake among Japanese schoolchildren: Current status and possible intervention to prevent excess intake. Public Health Nutr..

[69] Murakami K., Okubo H., Livingstone M.B.E., Fujiwara A., Asakura K., Uechi K., Sugimoto M., Wang H.-C., Masayasu S., Sasaki S. (2018). Adequacy of Usual Intake of Japanese Children Aged 3–5 Years: A Nationwide Study. Nutrients.

[70] Yokoyama T. (2013). Theory and Application of Statistical Methods to Estimate the Distribution of Usual Intakes of a Nutrient in a Population: For the Appropriate Use of Dietary Reference Intakes. Jpn. J. Nutr. Diet..

[71] Dodd K.W., Guenther P.M., Freedman L.S., Subar A.F., Kipnis V., Midthune D., Tooze J.A., Krebs-Smith S.M. (2006). Statistical Methods for Estimating Usual Intake of Nutrients and Foods: A Review of the Theory. J. Am. Diet. Assoc..

[72] Nusser S., Carriquiry A., Dodd K., Fuller W. (1996). A semiparametric transformation approach to estimating usual daily intake distributions. J. Am. Stat. Assoc..

[73] Ministry of Health, Labour and Welfare of Japan (2015). Dietary Reference Intakes for Japanese. http://www.mhlw.go.jp/stf/seisakunitsuite/bunya/0000208970.html.

[74] Azaïs-Braesco V., Sluik D., Maillot M., Kok F., Moreno L.A. (2017). A review of total & added sugar intakes and dietary sources in Europe. Nutr. J..

[75] Guelinckx I., Ferreira-Pêgo C., Moreno L.A., Kavouras S.A., Gandy J., Martinez H., Bardosono S., Abdollahi M., Nasseri E., Jarosz A. (2015). Intake of water and different beverages in adults across 13 countries. Eur. J. Nutr..

[76] Guelinckx I., Iglesia I., Bottin J.H., De Miguel-Etayo P., González-Gil E.M., Salas-Salvadó J., Kavouras S.A., Gandy J., Martinez H., Bardosono S. (2015). Intake of water and beverages of children and adolescents in 13 countries. Eur. J. Nutr..

[77] O’Neil C.E., Keast D.R., Fulgoni V.L., Nicklas T.A. (2012). Food sources of energy and nutrients among adults in the US: NHANES 2003-2006. Nutrients.

[78] Sluik D., van Lee L., Engelen A.I., Feskens E.J.M. (2016). Total, Free, and Added Sugar Consumption and Adherence to Guidelines: The Dutch National Food Consumption Survey 2007-2010. Nutrients.

[79] Sette S., Le Donne C., Piccinelli R., Mistura L., Ferrari M., Leclercq C. (2013). The third National Food Consumption Survey, INRAN-SCAI 2005–06: Major dietary sources of nutrients in Italy. Int. J. Food Sci. Nutr..

[80] Gibson S., Francis L., Newens K., Livingstone B. (2016). Associations between free sugars and nutrient intakes among children and adolescents in the UK. Br. J. Nutr..

[81] Lei L., Rangan A., Flood V.M., Louie J.C.Y. (2016). Dietary intake and food sources of added sugar in the Australian population. Br. J. Nutr..

[82] Ministry of Health, Labour and Welfare of Japan (2016). Summary of Situation Related to Daycare Centers. http://www.mhlw.go.jp/stf/houdou/0000176137.html.

[83] Ministry of Education, Culture, Sports, Science and Technology of Japan (2014). School Health Statistics Survey. http://www.mext.go.jp/component/b_menu/other/__icsFiles/afieldfile/2015/03/27/1356103_3.pdf.

[84] Ministry of Health, Labour and Welfare of Japan (2010). National Growth Survey on Preschool Children. http://www.mhlw.go.jp/toukei/list/73-22.html.

[85] Lanigan J.A., Wells J.C.K., Lawson M.S., Cole T.J., Lucas A. (2004). Number of days needed to assess energy and nutrient intake in infants and young children between 6 months and 2 years of age. Eur. J. Clin. Nutr..

[86] Sasaki S., Takahashi T., Iitoi Y., Iwase Y., Kobayashi M., Ishihara J., Akabane M., Tsugane S. (2003). Food and Nutrient Intakes Assessed with Dietary Records for the Validation Study of a Self-administered Food Frequency Questionnaire in JPHC Study Cohort l. J. Epidemiol..

[87] Tani Y., Asakura K., Sasaki S., Hirota N., Notsu A., Todoriki H., Miura A., Fukui M., Date C. (2015). The influence of season and air temperature on water intake by food groups in a sample of free-living Japanese adults. Eur. J. Clin. Nutr..

[88] Livingstone M.B.E., Black A.E. (2003). Markers of the validity of reported energy intake. J. Nutr..

[89] Yasui T., Furukawa T., Hase S. (1980). High Performance Liquid Chromatographic Determination of Saccharides in Dairy Products. Nippon Shokuhin Kogyo Gakkaishi.

[90] Tsuji M., Komiyama Y. (1986). Sugar composition and water activity of confectionary in Yamanashi prefecture and the other prefecture. Rep. Inst. Wine Food Technol. Yamanashi Prefect..

[91] Ishiguro K., Date Y. (1980). Sugars Content of Drinks and Ice-Cakes on Market: Separative Determination of Glucose, Fructose and Sucrose by Gaschromctography. Jpn. J. Nutr. Diet..

[92] Greenfield H., Southgate D. (2003). Food Composition Data: Production, Management and Use.

[93] Yasui A. (2016). Outline of Standard Tables of Food Composition in Japan 2015 (Seventh Revised Edition). Jpn. J. Nutr. Diet..

[94] US Department of Agriculture USDA Database for the Added Sugars Content of Selected Foods, Release 1. https://www.ars.usda.gov/northeast-area/beltsville-md-bhnrc/beltsville-human-nutrition-research-center/nutrient-data-laboratory/docs/usda-database-for-the-added-sugars-content-of-selected-foods-release-1/.

[95] Hase S., Kawamura S., Tada M., Yoneyama S., Kanaya K. (1980). Studies on Determination of Starch in Agricultural Products Part 3 Cross-Check of the Enzymatic Method for Starch Determination. Rep. Natl. Food Res. Inst..

[96] National Food Institute Technical University of Denmark Frida Food Data Version 2. https://frida.fooddata.dk/index.php?lang=en.

