# Supplementary material for: Estimation of Starch and Sugar Intake in a Japanese Population Based on a Newly Developed Food Composition Database
Source: Nutrients. 2018 Oct 10;10(10):1474. doi: 10.3390/nu10101474 (PMC6213530; doi:10.3390/nu10101474)
Supplement: Supplementary file 1 [file nutrients-10-01474-s001.pdf]

Table S1 Number of food items in each step in the development of total sugar database

Food groups [a]	Steps [b]							Total
	1	2	3[c]	4	5	6	7	
Rice and grains	61	0	0	4	0	0	0	65
Bread	13	0	5	0	0	0	0	18
Noodle	43	0	0	4	0	0	0	47
Other grain products	29	0	8	3	1	0	0	41
Potatoes	44	4	2	0	0	0	5	55
Sugars and jams	30	0	0	0	6	0	0	36
Pulses and nuts	98	1	6	14	3	1	6	129
Vegetables	168	56	35	86	6	4	19	374
Mushrooms	46	3	0	1	0	0	0	50
Seaweeds	16	15	0	15	0	0	7	53
Fruits	58	0	39	13	1	8	8	127
Fish and shellfish	9	312	9	2	51	31	14	428
Meats	6	258	6	0	14	8	5	297
Eggs	18	1	0	0	1	0	0	20
Dairy products	36	2	0	1	0	3	0	42
Fats and oils	3	28	2	1	2	0	0	36
Confectionaries	132	0	20	5	6	0	0	163
Alcoholic beverages	11	8	2	6	2	3	1	33
Vegetable juices	2	0	2	2	0	0	0	6
Fruit juices	12	0	0	0	1	0	0	13
Tea and coffee	3	10	0	2	0	0	1	16
Sugar-sweetened beverages [d]	7	0	23	0	2	0	0	32
Seasonings	34	14	23	7	35	15	4	132
Other foods	1	0	6	0	2	0	0	9
Total	880	712	188	166	133	73	70	2222

[a] Food groups were defined based on the culinary usage and the similarity of nutrient profiles of the foods, mainly according to the Standard Tables of Food composition in Japan 2015 (STFCJ) [40,41].

[b] Step 1: assign values available in STFCJ [40,41]; step 2: assign 0 g per 100 g of food to foods with <1 g available carbohydrates (calculated as subtracting dietary fiber content (g per 100 g of food) from carbohydrate) per 100 g; step 3: assign analytical values reported in the literature; step 4: use values of similar foods available in STFCJ [40,41]; step 5: estimated based on recipes or ingredients; step 6: use values from food composition databases in other countries (the US [43], the UK [44], and Australia [45]); step 7: assign 0 g per 100 g of food.

[c] A literature search was conducted based on the PubMed, Ovid Medline, CiNii, and Ichushi-Web (a database for Japanese papers) databases, to identify studies that reported sugar content in Japanese foods. The search terms that were used included "(sugar OR sugars OR saccharide OR saccharides OR monosaccharide OR monosaccharides OR disaccharide OR disaccharides OR glucose OR fructose OR galactose OR sucrose OR maltose OR lactose OR trehalose) AND food AND (Japan OR Japanese)." Only papers that were written in English or Japanese were considered. References from relevant papers were also manually searched. Of 15 papers identified (seven based on high-performance liquid chromatography (HPLC) [48,49,52-54,89,90], one based on both HPLC and gas chromatography (GC) [55], four based on GC [56-58,91], and three based on enzymatic methods [50,51,59]), the papers in which data were obtained using HPLC were primarily considered; then, the papers in which data were obtained using GC or enzymatic methods were considered [92]. When multiple papers reported saccharide contents of the same food items using the same analytical methods, the value from the

newest paper was used, since the sugar contents in food items might change over time due to selective breeding, progress in cultivating techniques, and reformulation of commercial products [93,94]. Consequently, values reported in three [89,90,91] of the 15 candidate papers were not used. For each food item, the mean or median value was used as a representative value.

[d] Consisting of soda, sports drinks, fruit drinks, milk beverages, and pre-sweetened tea and coffee.

Table S2 Number of food items in each step in the development of starch database

Food groups [a]	Steps [b]								Total
	1	2	3[c]	4	5	6	7	8	
Rice and grains	61	0	0	4	0	0	0	0	65
Bread	13	0	0	0	4	0	1	0	18
Noodle	43	0	0	4	0	0	0	0	47
Other grain products	29	0	7	3	1	0	1	0	41
Potatoes	44	4	0	0	2	0	0	5	55
Sugars and jams	30	0	0	0	6	0	0	0	36
Pulses and nuts	98	1	0	14	3	1	6	6	129
Vegetables	168	56	5	98	6	3	19	19	374
Mushrooms	46	3	0	1	0	0	0	0	50
Seaweeds	16	15	0	15	0	0	0	7	53
Fruits	58	0	0	42	1	4	14	8	127
Fish and shellfish	9	312	0	2	53	31	7	14	428
Meats	6	258	2	0	20	6	2	3	297
Eggs	18	1	0	0	1	0	0	0	20
Dairy products	36	2	0	2	0	1	1	0	42
Fats and oils	3	28	0	1	2	0	2	0	36
Confectionaries	132	0	0	5	15	0	11	0	163
Alcoholic beverages	11	8	0	6	3	3	1	1	33
Vegetable juices	2	0	0	2	0	0	2	0	6
Fruit juices	12	0	0	0	1	0	0	0	13
Tea and coffee	3	10	0	2	0	0	0	1	16
Sugar-sweetened beverages [d]	7	0	0	18	2	0	5	0	32
Seasonings	34	14	5	13	35	1	26	4	132
Other foods	1	0	0	0	6	0	2	0	9
Total	880	712	19	232	161	50	100	68	2222

[a] Food groups were defined based on the culinary usage and the similarity of nutrient profiles of the foods, mainly according to the Standard Tables of Food composition in Japan 2015 (STFCJ) [40,41].
[b] Step 1: assign values available in STFCJ [40,41]; step 2: assign 0 g per 100 g of food to foods with <1 g available carbohydrates (calculated as subtracting dietary fiber content (g per 100 g of food) from carbohydrate) per 100 g; step 3: assign analytical values reported in the literature; step 4: use values of similar foods available in STFCJ [40,41]; step 5: estimated based on recipes or ingredients; step 6: use values from food composition databases in other countries (the US [43], the UK [44], and Australia [45]) ; step 7: assign values calculated as subtracting total sugar contents from available carbohydrate contents (calculated as subtracting dietary fiber contents from carbohydrate contents); step 8: assign 0 g per 100 g of food.
[c] A literature search was conducted based on the PubMed, Ovid Medline, CiNii, and Ichushi-Web (a database for Japanese papers) databases, to identify studies that reported starch contents in Japanese foods using the search terms "(starch OR starches) AND food AND (Japan OR Japanese)." In total, three papers were identified: two based on enzymatic methods [60, 95] and one based on Somogyi methods [61]. Since a part of the samples that were analysed in one paper [58] based on enzymatic methods were assumed to be analysed in another paper [95], values that were reported in two specific papers [60, 61] were used. For each food item, the median value was used as a representative value.
[d] Consisting of soda, sports drinks, fruit drinks, milk beverages, and pre-sweetened tea and coffee.

Table S3 Number of food items in each step in the development of free sugar database

Food groups [a]	Steps [b]											Total
	1	2	3	4	5	6	7	8	9	10	11	
Rice and grains	14	50	0	1	0	0	0	0	0	0	0	65
Bread	0	13	0	0	0	1	0	0	2	2	0	18
Noodle	7	33	0	0	0	0	0	7	0	0	0	47
Other grain products	2	28	2	0	0	0	0	8	1	0	0	41
Potatoes	14	35	0	2	0	0	0	3	1	0	0	55
Sugars and jams	0	0	27	5	0	0	1	2	1	0	0	36
Pulses and nuts	6	107	3	2	4	0	0	4	0	2	1	129
Vegetables	67	254	0	22	3	0	0	16	1	6	5	477
Mushrooms	14	34	0	0	2	0	0	0	0	0	0	50
Seaweeds	38	11	0	0	1	0	0	2	0	1	0	53
Fruits	0	101	10	0	3	0	0	13	0	0	0	127
Fish and shellfish	337	20	69	0	0	0	0	0	0	0	2	428
Meats	262	1	30	0	0	0	0	0	0	0	4	297
Eggs	1	14	0	2	0	0	0	0	3	0	0	20
Dairy products	7	32	0	0	0	3	0	0	0	0	0	48
Fats and oils	28	4	0	0	0	0	0	2	2	0	0	36
Confectionaries	4	6	103	22	0	13	0	3	10	2	0	163
Alcoholic beverages	10	15	0	1	0	0	2	5	0	0	0	33
Vegetable juices	0	6	0	0	0	0	0	0	0	0	0	6
Fruit juices	0	0	13	0	0	0	0	0	0	0	0	13
Tea and coffee	12	4	0	0	0	0	0	0	0	0	0	16
Sugar-sweetened beverages [c]	0	0	5	0	0	6	0	21	0	0	0	26
Seasonings	20	28	6	3	3	1	0	32	29	10	0	132
Other foods	0	0	0	2	0	0	0	1	2	4	0	9
Total	843	796	268	62	16	24	3	119	52	27	12	2222

[a] Food groups were defined based on the culinary usage and the similarity of nutrient profiles of the foods, mainly according to the Standard Tables of Food composition in Japan 2015 (STFCJ) [40,41].

[b] Free sugar contents were defined as follows: step 1: assign 0 g per 100 g of food to foods with 0 g total sugar per100 g; step 2: assign 0g per 100g of food to no free sugar food groups (e.g. plain cereals (such as grains, breads, pastas, rice, and flours), plain nuts and pulses, fresh fruits and vegetables, fresh meat and seafood, egg, non-sweetened dairy products, fats and oils, 100% vegetable juices, non-sweetened coffee and tea, and non-sweetened alcoholic beverages); step 3: assign values of total sugar to 100% free sugar food groups (e.g. sugar and syrups; processed meats; confectioneries not containing fruits, dairy products, and chocolates; soft drinks except for fruit drinks, 100% fruit juices, and bouillon cubes); step 4: calculated based on standard recipes available in STFCJ [40,41] to foods whose ingredients were all assigned in steps 1-3; step 5: calculated based on unsweetened variety; step 6: Estimated from content of each saccharide (i.e. calculated as subtracting lactose content from total sugar content for sweetened dairy products and confectionaries containing daily products); step 7: use values from food composition databases in other countries (Australia [43] (n 1) and Denmark [96] (n 2)); step 8: calculated based on common recipes or ingredients; step 9: calculated based on standard recipes available in STFCJ [40,41] for foods with ingredient values that were assigned in from steps 5-8; step 10: assign a half of total sugar content; step 11: assign 0 g per 100 g of food.

[c] Consisting of soda, sports drinks, fruit drinks, milk beverages, and pre-sweetened tea and coffee.

Table S4 Basic characteristic of Japanese children and adults

	Toddlers (Aged 18-35 months) [a]				Preschool children (Aged 3-6 years) [b]				Schoolchildren (Aged 8-14 years) [c]				Adults (Aged 20-69 years) [d]			
	**Boys (n = 183)**		**Girls (n = 185)**		**Boys (n = 186)**		**Girls (n = 190)**		**Boys (n = 435)**		**Girls (n = 480)**		**Men (n = 196)**		**Women (n = 196)**	
	**Mean**	**SD**	**Mean**	**SD**	**Mean**	**SD**	**Mean**	**SD**	**Mean**	**SD**	**Mean**	**SD**	**Mean**	**SD**	**Mean**	**SD**
Age (months or years) [e]	25.8	5.4	25.7	5.3	4.5	1.1	4.5	1.1	10.9	2.1	10.9	2.0	44.7	13.3	44.4	13.5
Height (cm) [f]	85.2	5.2	84.3	5.1	106.3	8.4	105.8	8.4	145.4	14.7	143.5	11.6	170.3	5.4	157.6	5.7
Weight (kg) [f]	12.1	1.6	11.6	1.5	17.8	3.4	17.8	3.5	40.1	13.7	38.1	10.8	69.6	11.3	56.1	10.0
BMI (kg/m^2) [g]	16.6	1.2	16.3	1.4	15.6	1.4	15.8	1.5	18.4	3.4	18.2	3.0	24.0	3.5	22.6	3.7
EI/EER [hi]	1.22	0.23	1.23	0.24	1.05	0.24	1.02	0.17	1.02	0.18	0.98	0.18	0.92	0.19	0.97	0.19

SD, standard deviation; BMI, body mass index; EI, energy intake; and EER, estimated energy requirement.

[a] 91-93 boys or girls for the each of the 18-23 months and 24-35 months.

[b] 44-48 boys or girls for the each of the 3, 4, 5, and 6 years.

[c] 135-176 boys or girls for each of the 2-year age groups (8–9, 10–11, and 13–14 years).

[d] 37-42 men or women for each of the 10-year age groups (20–29, 30–39, 40–49, 50–59, and 60–69 years).

[e] Months for toddlers and years for other participants.

[f] Measured to the nearest 0.1 cm (height) and 0.1 kg (weight) while the participants wearing light clothing and no shoes.

[g] Calculated by dividing body weight in kilograms by the squared body height in meters.

[h] For toddlers, EI was estimated using 1-day dietary data. For other participants, habitual EI was calculated based on the best-power method (performed with HabitDist [70]) by using 3-day dietary data for preschool children and schoolchildren or 4-day dietary data for adults [71, 72].

[i] Calculated as the ratio of reported EI to EER for each participant using the sex-and age-specific EER for medium level of physical activity in Dietary Reference Intakes for Japanese, 2015 [73]. EER (kcal/day) used was: 950 (for boys aged 18-35 months), 900 (for girls aged 18-35 months); 1300 (for boys aged 3-5 years), 1250 (for girls aged 3-5 years), 1550 (for boys aged 6 years), 1450 (for girls aged 6 years), 1850 (for boys aged 8-9 years), 1700 (for girls aged 8-9 years), 2250 (for boys aged 10-11 years), 2100 (for girls aged 10-11 years), 2600 (for boys aged 13-14 years), 2400 (for girls aged 13-14 years), 2650 (for men aged 20-49 years), 1950 (for women aged 20-29 years), 2000 (for women aged 30-49 years),2450 (for men aged 50-69 years), and 1900 (for women aged 50-69 years) [73].

Table S5 Estimated intakes of energy, maltose, lactose, trehalose, glucose, and galactose in Japanese children and adults

	Toddlers (Aged 18-35 months) [a]				Preschool children (Aged 3-6 years) [b]				Schoolchildren (Aged 8-14 years) [b]				Adults (Aged 20-69 years) [b]			
	**Boys (*n* = 183)**		**Girls (*n* = 185) [c]**		**Boys (*n* = 186)**		**Girls (*n* = 190) [c]**		**Boys (*n* = 435)**		**Girls (*n* = 480) [c]**		**Men (*n* = 196)**		**Women (*n* = 196) [c]**	
	**Mean**	**SD**	**Mean**	**SD**	**Mean**	**SD**	**Mean**	**SD**	**Mean**	**SD**	**Mean**	**SD**	**Mean**	**SD**	**Mean**	**SD**
**Absolute Value (kcal/day)**																
Energy	1126	208	1048***	206	1430	207	1320***	176	2257	453	2007***	296	2358	407	1888***	299
**Absolute Value (g/day)**																
Maltose	1.0	1.5	1.0	1.3	1.5	0.5	1.6	0.6	2.0	0.6	2.0	0.6	1.8	0.7	1.9	1.0
Lactose	10.8	6.4	11.2	5.8	10.3	4.0	9.0***	3.0	13.1	3.8	11.8***	2.1	5.3	4.4	6.3*	3.8
Trehalose	0.11	0.12	0.11	0.13	0.13	0.02	0.12	0.04	0.21	0.05	0.18***	0.06	0.20	0.10	0.21	0.09
Glucose	8.2	3.9	7.8	3.9	10.2	3.0	9.6*	2.4	12.8	3.5	11.8***	3.3	13.1	4.3	11.9**	3.8
Galactose	0.3	0.3	0.3	0.3	0.2	0.2	0.2	0.1	0.2	0.1	0.2	0.1	0.2	0.2	0.3**	0.2
**Energy-Adjusted Value (% of Energy)**																
Maltose	0.3	0.6	0.4	0.5	0.4	0.1	0.5**	0.2	0.4	0.1	0.4***	0.1	0.3	0.1	0.4***	0.2
Lactose	3.9	2.3	4.3	2.1	2.9	1.1	2.7	0.9	2.4	0.6	2.4	0.3	0.9	0.7	1.3***	0.8
Trehalose	0.04	0.04	0.04	0.05	0.04	0.01	0.04**	0.01	0.04	0.01	0.04*	0.01	0.03	0.02	0.04***	0.02
Glucose	2.9	1.2	3.0	1.3	2.9	0.7	2.9	0.6	2.3	0.5	2.4*	0.5	2.3	0.7	2.5***	0.7
Galactose	0.10	0.11	0.09	0.12	0.07	0.05	0.07	0.03	0.04	0.02	0.04	0.02	0.04	0.03	0.06***	0.05

SD, standard deviation.

[a] Estimated using 1-day dietary data.

[b] Habitual intake was calculated based on the best-power method (performed with HabitDist [70]) by using 3-day dietary data for preschool children and schoolchildren or 4-day dietary data for adults [71, 72].

[c] Significantly different from the corresponding male group determined by independent *t*-test for continuous variables and chi-square test for categorical variables; * P<0.05, ** P<0.01, *** P<0.001.

Table S6 Contribution (%) of each food group to intakes of maltose, lactose, trehalose, glucose, and galactose in Japanese children and adults [ab]

Nutrients	Food groups [cd]	Toddlers (Aged 18-35 months) (n = 368)	Preschool children (Aged 3-6 years) (n = 376)	Schoolchildren (Aged 8-14 years) (n = 915)	Adults (Aged 20-69 years) (n = 392)
Maltose	Bread	24.3	26.1	27.2	25.0
	Seasonings	19.5	14.2	17.9	20.9
	Confectionaries	18.0	23.3	17.7	12.2
	Potatoes	10.2	6.2	3.1	4.3
	Noodle	7.5	10.6	12.6	13.5
	Others	20.5	19.6	21.4	24.2
Lactose	Dairy products	83.8	67.1	68.2	38.0
	Sugar-sweetened beverages [e]	7.6	10.2	5.7	13.9
	Confectionaries	4.8	14.8	15.0	20.6
	Seasonings	2.8	4.7	6.6	18.0
	Others	1.1	3.2	4.5	9.5
Trehalose [f]	Mushrooms	69.4	55.9	61.6	50.4
	Bread	20.0	27.9	24.9	26.8
	Other grain products	5.3	5.3	4.9	8.2
	Noodle	3.5	10.0	6.4	12.4
	Others	1.8	0.9	2.2	2.2
Glucose	Fruits	27.3	21.0	10.3	10.5
	Vegetables	24.2	21.8	29.1	28.1
	Seasonings	17.4	17.4	24.4	25.1
	Sugar-sweetened beverages [e]	9.6	12.7	10.4	9.1
	Others	21.5	27.1	25.9	27.2
Galactose	Seasonings	49.5	53.7	71.0	67.3
	Dairy products	29.7	25.4	21.4	19.0
	Sugar-sweetened beverages [e]	19.1	16.4	3.9	9.3
	Others	1.7	4.5	3.7	4.4

[a] Values are means. The mean value of contribution was calculated by using 1-day dietary data (for toddlers) or the mean of 3- or 4-day dietary data (for other age groups).
[b] Only food groups with ≥7% contribution in at least one population or at least top three contributors are listed. Food groups with <7% contribution in all populations are not shown, and combined into others.

[c] Twenty-four food groups were defined based on the culinary usage and the similarity of nutrient profiles of the foods, mainly according to the Standard Tables of Food composition in Japan 2015 [40, 41]. They consisted of rice and grains, pulses and nuts, sugars and jams, seaweeds, fish and shellfish, meats, eggs, fat and oil, alcoholic beverages, vegetable juices, fruit juices, tea and coffee, and other foods in addition to the items listed in this table.
[d] Food groups are listed in descending order of the contribution in toddlers.
[e] Consisting of soda, sports drinks, fruit drinks, milk beverages, and pre-sweetened tea and coffee.
[f] n 320 for toddlers, n 373 for preschool children, n 913 for schoolchildren, and n 386 for adults due to the presence of nonconsumers.
